# A nationwide survey on non-B, non-C hepatocellular carcinoma in Japan: 2011–2015 update

**DOI:** 10.1007/s00535-018-1532-5

**Published:** 2018-11-29

**Authors:** Ryosuke Tateishi, Koji Uchino, Naoto Fujiwara, Tetsuo Takehara, Takeshi Okanoue, Masataka Seike, Hitoshi Yoshiji, Hiroshi Yatsuhashi, Masahito Shimizu, Takuji Torimura, Mitsuhiko Moriyama, Isao Sakaida, Hiroyuki Okada, Tetsuhiro Chiba, Makoto Chuma, Kazuhiko Nakao, Hajime Isomoto, Yutaka Sasaki, Shuichi Kaneko, Tsutomu Masaki, Kazuaki Chayama, Kazuhiko Koike

**Affiliations:** 1 Department of Gastroenterology, Graduate School of Medicine, The University of Tokyo, 7-3-1 Hongo, Bunkyo-Ku, Tokyo, 113-8655 Japan; 2 Department of Gastroenterology and Hepatology, Osaka University Graduate School of Medicine, Suita, Japan; 3 Department of Gastroenterology and Hepatology, Saiseikai Suita Hospital, Suita, Japan; 4 Depatment of Gastroenterology, Faculty of Medicine, Oita University, Yufu, Japan; 5 Third Department of Internal Medicine, Nara Medical University, Kashihara, Japan; 6 Clinical Research Center, National Hospital Organization (NHO) Nagasaki Medical Center, Nagasaki University Graduate School of Biomedical Sciences, Nagasaki, Japan; 7 Department of Gastroenterology, Gifu University Graduate School of Medicine, Gifu, Japan; 8 Division of Gastroenterology, Department of Internal Medicine, Kurume University School of Medicine, Kurume, Japan; 9 Division of Gastroenterology and Hepatology, Department of Internal Medicine, Nihon University School of Medicine, Tokyo, Japan; 10 Department of Gastroenterology and Hepatology, Yamaguchi University Graduate School of Medicine, Ube, Japan; 11 Department of Gastroenterology and Hepatology, Okayama University Graduate School of Medicine, Dentistry and Pharmaceutical Sciences, Okayama, Japan; 12 Department of Gastroenterology and Nephrology, Graduate School of Medicine, Chiba University, Chiba, Japan; 13 Gastroenterological Center, Yokohama City University Medical Center, Yokohama, Japan; 14 Department of Gastroenterology and Hepatology, Nagasaki University of Graduate School of Biomedical Sciences, Nagasaki, Japan; 15 Faculty of Medicine, Medicine and Clinical Science, Tottori University, Yonago, Japan; 16 Department of Gastroenterology and Hepatology, Graduate School of Medical Sciences, Kumamoto University, Kumamoto, Japan; 17 Department of Gastroenterology, Graduate School of Medicine, Kanazawa University, Kanazawa, Japan; 18 Department of Gastroenterology and Neurology, Faculty of Medicine, Kagawa University, Takamatsu, Japan; 19 Department of Gastroenterology and Metabolism, Graduate School of Biomedical and Health Sciences, Hiroshima University, Hiroshima, Japan

**Keywords:** Hepatocellular carcinoma, Non-alcoholic fatty liver disease, Alcoholic liver disease

## Abstract

**Background:**

We previously reported that the incidence of hepatocellular carcinoma (HCC) with non-viral etiologies increased rapidly between 1991 and 2010 in Japan.

**Methods:**

To update this investigation, we enrolled patients who were initially diagnosed as having non-B, non-C HCC at participating hospitals between 2011 and 2015. In addition to the patient characteristics investigated in the previous report, we also investigated the duration of alcohol consumption. The overall survival rate was analyzed using the Kaplan–Meier method, and the hazard function against the body mass index (BMI) was plotted using cubic splines.

**Results:**

A total of 2087 patients were enrolled. The proportion of patients with non-viral etiologies has continued to increase from 10.0% in 1991 to 32.5% in 2015. Patients were also older (median ages, 70–73 years) and more obese (median BMIs, 23.9–24.2 kg/m^2^), and the proportions of patients with diabetes mellitus (46.1% to 51.6%), hypertension (42.7% to 58.6%), dyslipidemia (14.6% to 22.9%), and fatty liver (24.0% to 28.8%) had all increased significantly. There was a significant inverse relationship between the duration and the amount of daily alcohol consumption. The improvement in the overall survival was relatively small, with a decreased proportion of patients under surveillance (41.3% to 31.6%). A hazard function plot showed a curve similar to that in our previous report, with a lowest hazard of ~ 26 kg/m^2^.

**Conclusions:**

The proportion of HCC patients with non-viral etiologies continues to increase in Japan. Lifetime total amount of alcohol consumption may be a risk factor.

**Electronic supplementary material:**

The online version of this article (10.1007/s00535-018-1532-5) contains supplementary material, which is available to authorized users.

## Introduction

Chronic hepatitis B and C has contributed to primary liver cancer, especially the development of hepatocellular carcinoma (HCC), in the vast majority of such cases worldwide [1]. As a result of efforts to control hepatitis B (HBV) and hepatitis C virus (HCV) infections, mortality rates have started to decrease in Asia and Southern Europe [2]. On the other hand, the mortality rates continue to increase in other regions such as the United States, where the increase in the incidence of HCC was the highest among all types of cancer between 2003 and 2012 [3].

Growing evidence suggests that obesity and diabetes increase various cancer risks [4–8], with obesity and obesity-associated conditions having the largest impact on carcinogenic processes in the liver [5, 6, 9, 10]. In fact, the population-attributable fractions of obesity and diabetes on liver cancer development were the largest among various risk factors in the United States, suggesting that the diversity in obese populations among races and ethnicities has contributed to the disparity in the increasing rates of HCC in the United States [11].

We previously reported that the incidence of HCC with a non-viral etiology increased rapidly between 1991 and 2010 and that the proportion of patients with obesity or diabetes was higher than that of patients with viral hepatitis during this period. We concluded that the increase in the obese population among Japanese males over the last three decades has contributed to the increase in HCC patients with a non-viral etiology [12]. The present study aims to update this investigation by collecting data from 2011 to 2015 and by including additional information regarding detailed histories of alcohol consumption and prescriptions for diabetes, hypertension, and dyslipidemia.

## Patients and methods

### Study design

This retrospective study complied with the ethical guidelines for medical and health research involving human subjects established by the Japanese Ministry of Education, Culture, Sports, Science, and Technology and the Ministry of Health, Labour, and Welfare. The study protocol was approved by the University of Tokyo Medical Research Center Ethics Committee (approval number 3710) and the Institutional Review Board or Ethics Committee of each participating institution. Informed consent was waived because of the retrospective design. This study was registered with the University Hospital Medical Information Network (UMIN) Clinical Trial Registry (UMIN-CTR000007570).

### Patients

In the current study, we collected data from patients who were initially diagnosed as having non-B, non-C HCC at the participating hospitals between 2011 and 2015. Patients who were negative for both hepatitis B surface antigen (HBsAg) and anti-HCV antibody were enrolled. We also investigated the number of HCC patients who were initially diagnosed during the same period according to the following four categories: HBV single positive, HCV single positive, both HBV and HCV positive, and non-B, non-C. We also used data from our previous study, which had enrolled non-B, non-C HCC patients diagnosed between 1991 and 2010, for comparison purposes [12].

### Diagnosis of HCC

HCC was diagnosed pathologically or using imaging criteria based on the Japanese Clinical Practice Guidelines; hyperattenuation during the arterial phase with washout during the late phase on dynamic CT or dynamic MRI images was regarded as a specific finding [13].

### Data collection

The patients were registered via an electronic data capture system designed by the investigators. We collected data at the time of the initial diagnosis of HCC; this data included anthropometric parameters, daily alcohol consumption, medical comorbidities, tumor characteristics, treatment modalities and laboratory data. The collected items were described in detail in our previous report [12]. The body mass index (BMI), Child–Turcotte–Pugh (CTP) score [14], fibrosis-4 (FIB-4) index [15], and Barcelona Clinic Liver Cancer (BCLC) stage [16] were calculated automatically using the obtained data.

The etiologies of the background liver diseases were categorized as follows: autoimmune hepatitis (AIH), primary biliary cholangitis (PBC), AIH-PBC overlap syndrome, alcoholic liver disease (ALD), non-alcoholic fatty liver disease (NAFLD), Budd–Chiari syndrome, hemochromatosis, Wilson’s disease, and others. Diagnoses of background liver diseases, diabetes mellitus, hypertension, and dyslipidemia were made by the attending physician based on the Japanese clinical guidelines for each disease. Daily alcohol consumption was calculated from reported forms regarding alcohol intake and frequency. Regarding the diagnosis of alcoholic liver disease, we used previously reported criteria of a daily alcohol consumption ≥ 80 g/day and the absence of any other definite etiology of liver disease in our previous report. In the present report, however, we revised the criterion for alcohol consumption to ≥ 60 g/day based on the Japanese Society for Biomedical Research on Alcohol (JASBRA) Diagnostic Criteria for Alcoholic Liver Disease 2011 Edition [17]. The background etiologies in the 1991–2010 survey were recalculated based on this new criterion. NAFLD was defined as the presence or a history of fatty liver diagnosed radiologically or pathologically and an alcohol consumption of ≤ 20 g/day.

The survival statuses of the patients were also registered. Survival status was defined as alive, dead, or lost to follow-up. Observations were censored as of December 31, 2015. For the deceased patients, the cause of death was categorized according to the criteria of the Liver Cancer Study Group of Japan [18] as follows: liver cancer progression, liver failure, gastrointestinal bleeding, gastroesophageal varices rupture, rupture of liver cancer, operative death, other, or unknown.

In addition to the data investigated in our previous report, we also collected the following data in the present study. First, we investigated the history of fatty liver. Second, we investigated the ages at which the patients started and stopped drinking and whether the patients had practiced moderation in their drinking for at least 6 months prior to the diagnosis of HCC. Moderation in drinking was defined as decreased amount of daily alcohol consumption from the peak value. We also compared the γ-glutamyltransferase (GGT), aspartate aminotransferase (AST), and alanine aminotransferase (ALT) values and the FIB-4 index between patients who had practiced moderation in their drinking for longer than 6 months prior to the diagnosis of HCC and patients who had continued to drink, matched for gender and daily amount of alcohol intake. Finally, we investigated the presence of prescriptions for the treatment of diabetes, hypertension, and dyslipidemia.

### Statistical analysis

Data are expressed as the medians with the 25th to 75th percentiles unless otherwise indicated. Numbers and percentages were used for qualitative variables. The Student’s *t* test or the Wilcoxon rank-sum test was used for comparisons between two continuous variables. For the comparison of paired data, the Wilcoxon signed-rank test was used. Differences among groups were assessed using a one-way analysis of variance (ANOVA) for continuous data and the Chi-squared test for categorical data. The Cochran–Armitage trend test was used to evaluate increasing or decreasing trends. Survival time was defined as the interval between the day of the initial diagnosis and death or the last visit to the hospital. Cumulative survival curves were constructed using the Kaplan–Meier method and were compared using the log-rank test. To assess the hazard ratios of various factors on overall survival, the Cox proportional hazard model was used. We also plotted the relative hazard against BMI using cubic splines.

All the statistical analyses were performed using R software (ver. 3.2.3; R Development Core Team, Vienna, Austria). All the tests were two-sided, and *P* values < 0.05 were considered to indicate statistical significance.

## Results

### Patient profiles

**Fig. 1:**
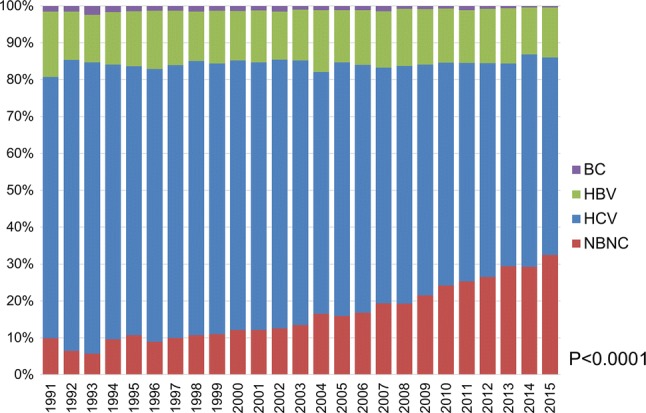
Trend in background liver diseases among patients with hepatocellular carcinoma in Japan. The current and previous cohorts were combined. A marked increase in the proportion of patients categorized as non-B, non-C was observed at the participating hospitals (*P *< 0.0001, Cochran–Armitage test)

Thirty-four hospitals consented to participate in the current survey. Of the 7,370 patients who were initially diagnosed as having HCC at the participating hospitals, 2087 (28.3%) were categorized as non-B, non-C. The proportion of patients with a non-B, non-C etiology continued to increase in 2011–2015 (Fig. 1; *P *< 0.001, Cochran–Armitage test). The proportion of non-B, non-C patients increased from 10.0% in 1991 to 32.5% in 2015. The distribution of background liver diseases among non-B, non-C patients was as follows: AIH in 54 (2.6%), PBC in 52 (2.5%), AIH-PBC overlap syndrome in 10 (0.5%), alcoholic liver disease in 675 (32.3%), NAFLD in 315 (15.1%), Budd-Chiari Syndrome in 1 (0.04%), hemochromatosis in 2 (0.1%), Wilson’s disease in 4 (0.2%), unclassified in 911 (43.7%), and other in 63 (3.0%).

We compared the baseline characteristics of the current study cohort with the previous cohort (Table 1). The age at the time of diagnosis increased significantly, whereas the proportion of males was almost the same. The BMI increased slightly, whereas the proportions of patients with diabetes mellitus, hypertension, dyslipidemia, and fatty liver increased significantly. Although HCC was diagnosed at a slightly earlier stage in the current cohort, the difference was quite small.

**Table 1:** Baseline characteristics of HCC patients

	2011–2015	1991–2010	*P* value
Number of patients	2087	5326	
Etiology			< 0.0001
AIH	54 (2.6)	161 (3.0)	
PBC	52 (2.5)	166 (3.1)	
Alcoholic liver disease	675 (32.3)	1997 (37.5)	
NAFLD	315 (15.1)	596 (11.2)	
Unclassified	911 (43.7)	2301 (43.2)	
Others	80 (3.8)	105 (2.0)	
Age, year	73.0 (66.0–78.0)	70.0 (63.0–75.0)	< 0.0001
Male gender, *n* (%)	1560 (74.7)	4022 (75.5)	0.51
BMI (kg/m^2^)	24.2 (21.7–27.0)	23.9 (21.6–26.6)	0.003
Alcohol consumption (g/day)^a^			0.53
≤ 20, *n* (%)	1058 (50.7)	2623 (50.9)	
21–59, *n* (%)	263 (12.6)	602 (11.7)	
≥ 60, *n* (%)	766 (36.7)	1928 (37.4)	
Diabetes, *n* (%)^b^	1072 (51.6)	2345 (46.1)	< 0.0001
Hypertension, *n* (%)^c^	1201 (58.6)	2063 (42.7)	< 0.0001
Dyslipidemia, *n* (%)^d^	448 (22.9)	720 (14.6)	< 0.0001
Fatty liver, *n* (%)^e^	502 (28.8)	936 (24.0)	< 0.0001
Anti-HBc Ab positive, *n* (%)^f^	618 (35.3)	1501 (40.3)	< 0.0001
ALT (U/L)	30 (21–46)	32 (22–50)	< 0.0001
Platelet count (×10^9^/μL)^g^	14.0 (9.70–19.50)	135 (90–193)	0.002
FIB-4 index^h^	4.15 (2.56–6.50)	4.06 (2.50–6.71)	0.96
Child–Pugh class^i^			0.005
A, *n* (%)	1445 (69.7)	3500 (69.0)	
B, *n* (%)	530 (25.6)	1231 (24.3)	
C, *n* (%)	97 (4.7)	338 (6.7)	
Tumor characteristics			0.49
Maximal tumor size (cm)^j^	3.1 (2.0–6.2)	3.2 (2.0–6.0)	
Number of nodules^k^			0.019
Single, *n* (%)	1139 (54.6)	2700 (51.1)	
2–3, *n* (%)	488 (23.4)	1368 (25.9)	
> 3, *n* (%)	460 (22.0)	1220 (23.1)	
Vascular invasion^l^, *n* (%)	53 (2.5)	187 (3.5)	0.036
Extrahepatic metastasis^m^, *n* (%)	189 (9.1)	401 (7.6)	0.038
AFP^n^ (ng/mL)			
≤ 20, *n* (%)	1271 (62.0)	2908 (59.4)	0.016
21–200, *n* (%)	357 (17.4)	820 (16.8)	
> 200, *n* (%)	423 (20.6)	1164 (23.8)	
DCP^o^ (mAU/mL)			
≤ 100, *n* (%)	949 (47.0)	2121 (45.8)	
101–400, *n* (%)	308 (37.8)	787 (17.0)	
> 400, *n* (%)	764 (37.8)	1727 (37.3)	
AFP-L3^p^ (%)			< 0.0001
≤ 10, *n* (%)	832 (59.2)	1765 (67.7)	
10.1–15, *n* (%)	99 (7.0)	74 (2.8)	
> 15, *n* (%)	475 (33.8)	767 (29.4)	
BCLC stage^q^			< 0.0001
0/A, *n* (%)	1083 (51.9)	26n (49.6)	
B, *n* (%)	703 (33.7)	2023 (38.3)	
C, *n* (%)	207 (9.9)	312 (5.9)	
D, *n* (%)	94 (4.5)	329 (6.2)	

Data are expressed as the median (25th–75th percentiles) or number (percentages)

*AFP* alpha-fetoprotein, *AFP-L3 Lens culinaris* agglutinin-reactive fraction of AFP, *AIH* autoimmune hepatitis, *ALT* alanine aminotransferase, *Anti-HBc Ab* anti-hepatitis B core antibody, *BCLC* Barcelona Clinic Liver Cancer, *BMI* body mass index, *DCP* des-gamma-carboxy prothrombin, *FIB-4* fibrosis-4

Since only a few patients were categorized as having AIH-PBC overlap syndrome, Budd-Chiari syndrome, hemochromatosis and Wilson’s disease, these categories were combined with ‘others’. Data were missing for ^a^173, ^b^241, ^c^498, ^d^388, ^e^1434, ^f^1606, ^g^61, ^h^142, ^i^257, ^j^42, ^k^38, ^l^28, ^m^26, ^n^434, ^o^691, ^p^3677, and ^q^40 patients in the 1991-2010 cohort and ^b^10, ^c^36, ^d^128, ^e^346, ^g^312, ^i^15, ^n^36, ^o^66, and ^p^681 patients in the 2011-2015 cohort, respectively

Regarding the HCC diagnostic process, liver nodules were initially pointed out in 18.5% of the patients at the participating departments, and 31.6% were followed with imaging modalities before diagnosis. The proportion of patients under surveillance decreased, compared with that in the previous study (41.3%).

### Fatty liver and detailed history of alcohol consumption

Fatty liver was observed in 502 patients (28.8%) at the time of the diagnosis of HCC. Of the 1,656 patients in whom a previous history of fatty liver disease had been documented, fatty liver was observed in 449 (27.1%) prior to the diagnosis of HCC. Of these patients, 417 (92.9%) still exhibited fatty liver at the time of the diagnosis of HCC, whereas the fatty liver had disappeared in 32 (7.1%).

**Fig. 2:**
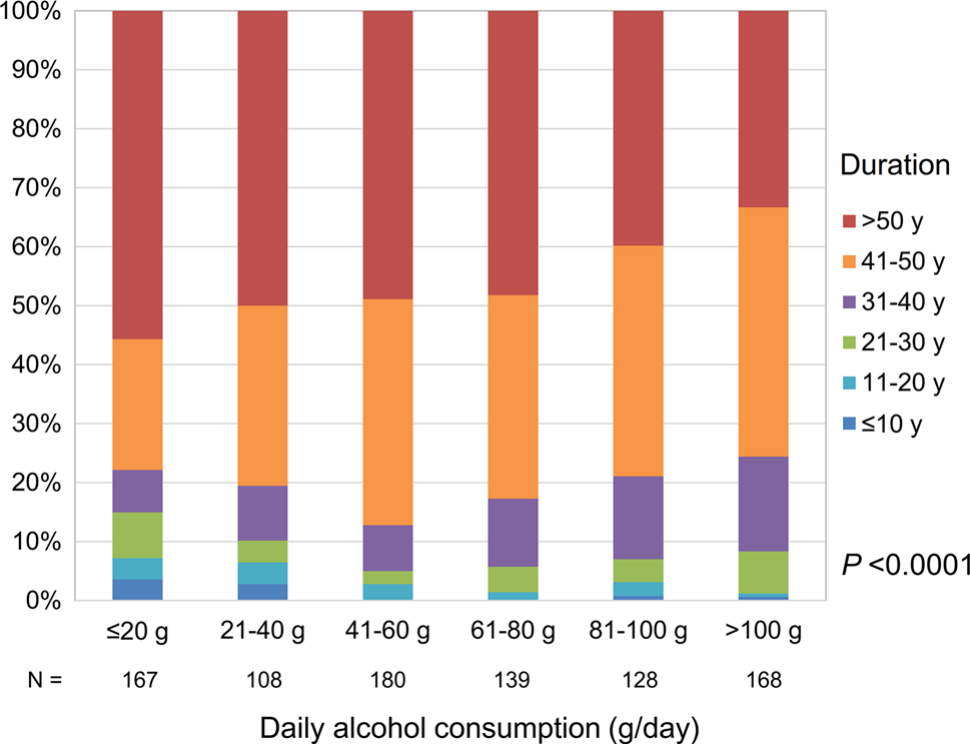
Duration and daily amount of alcohol consumption. Those who consumed a larger amount of alcohol daily tended to require a shorter duration of drinking to develop hepatocellular carcinoma (*P *< 0.0001, Cochran–Armitage test)

Regarding alcohol consumption, 1503 (72.0%) patients were former or current drinkers. Of them, the duration of drinking was available in 890. A significant inverse relationship between the duration of drinking and the amount of daily alcohol consumption was observed (Fig. 2, *P *< 0.0001, Cochran-Armitage test). The presence or absence of moderation in drinking prior to the diagnosis of HCC was known for 1,181 of the 1,503 patients. A total of 414 patients had practiced moderation in drinking, and we selected another group of patients without moderation in drinking by matching the groups for sex and daily amount of alcohol consumption in a 1:1 ratio. Consequently, 311 patients were retrieved in each group. The baseline characteristics of the patients are shown in Table 2. The differences in values between the two groups were most prominent for GGT, followed by AST; the difference in the ALT values was much smaller, and no significant difference in the FIB-4 indices was seen (Supplementary Fig. 1).

**Table 2:** Baseline characteristics of patients who practiced moderation in drinking and patients who continued drinking

	Moderation in drinking	Continuous drinking	*P* value
Number of patients	311	311	
Age, year	73.0 (66.0–78.0)	72.0 (65.0–77.0)	0.36
Male gender, *n* (%)	273 (87.8)	273 (87.8)	1
BMI (kg/m^2^)	24.3 (22.0–26.7)	23.9 (21.6–26.4)	0.64
Alcohol consumption (g/day)			1
≤ 20, *n* (%)	106 (34.1)	106 (34.1)	
21–59, *n* (%)	47 (15.1)	47 (15.1)	
≥ 60, *n* (%)	158 (50.8)	158 (50.8)	
Albumin (g/dL)	3.7 (3.3–4.2)	3.8 (3.4–4.2)	0.16
Platelet count (× 10^9^/μL)	13.8 (9.2–19.4)	14.0 (9.7–19.2)	0.57

*BMI* body mass index

### Medication for diabetes, hypertension, and dyslipidemia

Regarding medications for diabetes, dipeptidyl peptidase-4 inhibitors were the most common, followed by sulfonylurea. The proportion of patients using insulin was 23.3%. Calcium channel blockers were the most common anti-hypertensive medications, followed by angiotensin II receptor blockers. Statins were used in the vast majority of patients with dyslipidemia (Supplementary Fig. 2).

### Treatment and survival

The distribution of initial treatments was as follows: resection in 452 (21.7%), ablation in 394 (18.9%), ablation + TACE in 187 (9.0%), TACE in 656 (31.4%), transarterial chemotherapy with one-shot and continuous infusion in 146 (7.0%), systemic therapy in 58 (2.8%), radiation therapy in 24 (1.1%), liver transplantation in 8 (0.4%), others in 33 (1.6%), and supportive care in 129 (6.2%). The distribution was quite similar to the previous report.

**Fig. 3:**
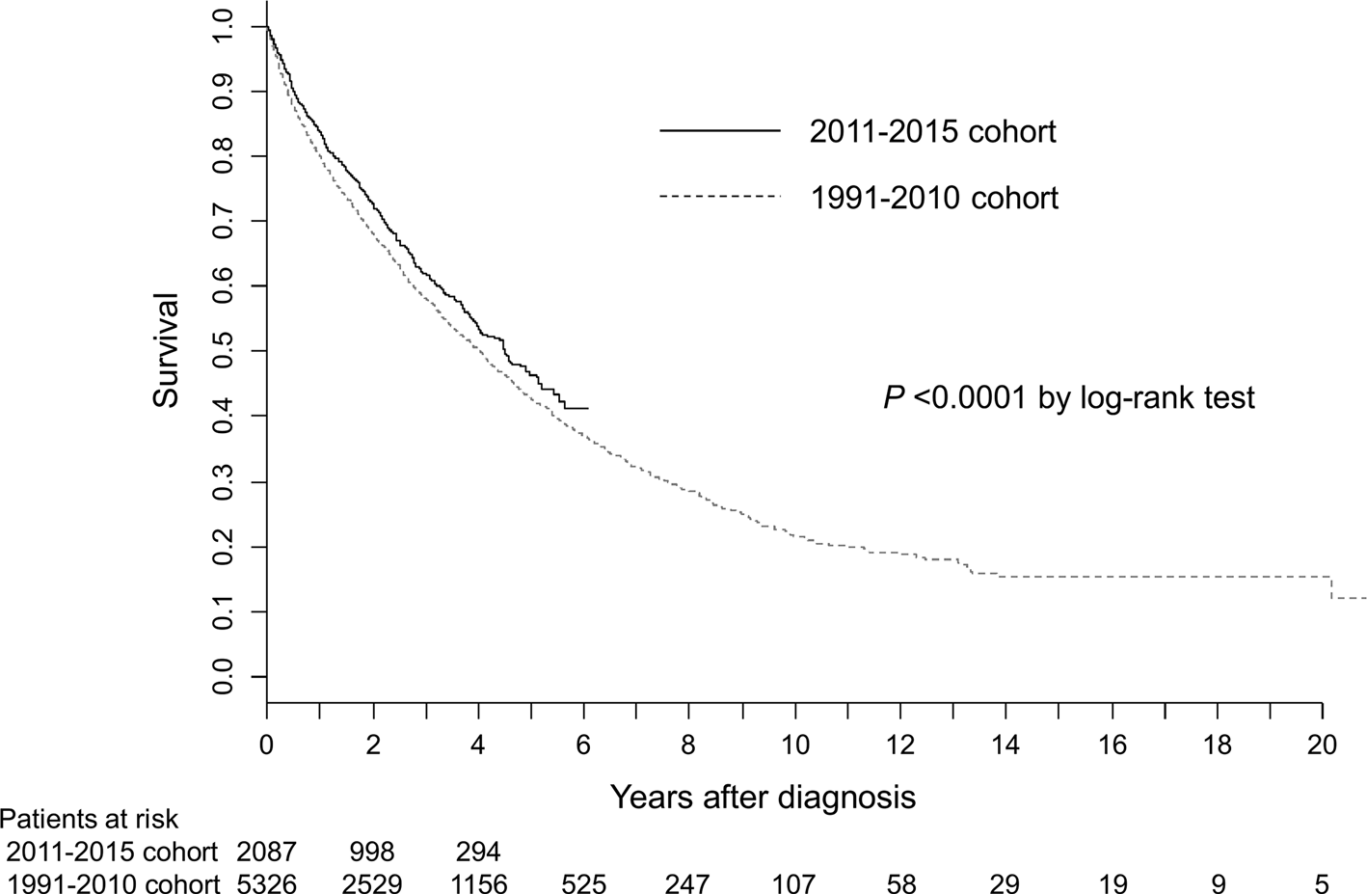
Overall survival of 2011–2015 cohort (solid line) and 1991–2010 cohort (dotted line)

**Fig. 4:**
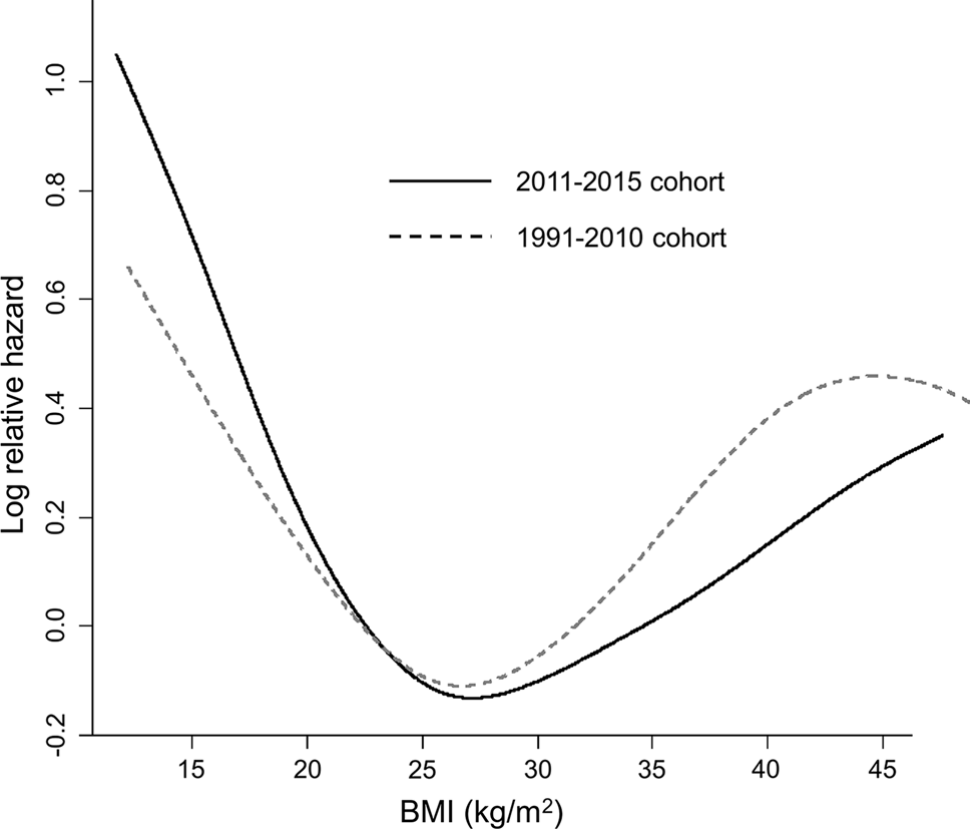
Estimated hazard function of BMI. The relative hazard was lowest at a BMI of ~ 26 kg/m^2^ for both the 2011–2015 cohort (solid line) and the 1991–2010 cohort (dotted line)

During the mean follow-up period of 2.1 years, 704 patients died and 272 patients were lost to follow-up. The causes of death were cancer progression in 465 (66.1%), liver failure in 63 (8.9%), gastrointestinal bleeding (including varices rupture) in 22 (3.1%), tumor rupture in 18 (2.6%), and others in 92 (13.1%). The cause of death was unspecified in 44 (6.3%). The median survival time (95% confidence interval [CI]) after the initial diagnosis of HCC was 4.52 (4.05–5.11) years. The overall survival rates at 1, 2, 3, and 5 years were 83.9, 72.6, 61.9, and 46.4%, respectively (Fig. 3). The survival was slightly improved, compared with the previous report (*P* < 0.0001, log-rank test). The hazard ratio of the new cohort versus the previous cohort was 0.898 (95% CI, 0.819–0.985) in the multivariate analysis using age, gender, and tumor- and liver function-related factors (Table 3). The estimated hazard plot according to the BMI exhibited J-shaped curves in both the current and previous cohorts (Fig. 4). The shape of the curves was quite similar to the previous report, with the lowest hazard at around 26 kg/m^2^.

**Table 3:** Multivariate Cox regression analysis of risk factors for overall survival

Variable	HR (95% CI)	*P* value
Cohort 2011–2015 vs. 1991–2010	0.896 (0.817–0.983)	0.02
Age per year	1.02 (1.02–1.03)	< 0.0001
Female vs. male	0.816 (0.729–0.913)	0.0004
Alcohol consumption (g/day)		
≤ 20	1	
21–59	1.02 (0.907–1.15)	0.72
≥ 60	1.03 (0.928–1.15)	0.59
BMI (kg/m^2^)		
< 18.5	1.35 (0.739–1.13)	0.0009
18.5–21.9	1.23 (1.10–1.37)	0.0003
22.0–24.9	1	
25.0–29.9	1.10 (0.986–1.22)	0.09
≥ 30.0	1.23 (1.05–1.45)	0.01
Maximal tumor (cm)		
≤ 2.0	1	
2.1–3.0	1.11 (0.973–1.28)	0.12
3.1–5.0	1.30 (1.14–1.49)	0.0001
5.1–10.0	1.94 (1.69–2.22)	< 0.0001
> 10.0	2.97 (2.53–3.48)	< 0.0001
Tumor number		
Single	1	
2–3	1.39 (1.26–1.55)	< 0.0001
4–5	1.80 (1.54–2.11)	< 0.0001
≥ 6	2.42 (2.15–2.74)	< 0.0001
Child–Pugh score (per 1 point)	1.37 (1.34–1.40)	< 0.0001
Extrahepatic metastasis, present	1.83 (1.60–2.09)	< 0.0001
Vascular invasion, present	1.57 (1.27–1.95)	< 0.0001
AFP (ng/mL)		
< 20	1	
20–99	1.47 (1.30–1.66)	< 0.0001
100–399	1.97 (1.70–2.28)	< 0.0001
≥ 400	2.22 (1.99–2.48)	< 0.0001

*AFP* alpha-fetoprotein

## Discussion

In the present study, we confirmed that the proportion of patients with a non-viral etiology continues to increase in Japan, as reported in our previous study. The numbers of patients with diabetes mellitus, hypertension, dyslipidemia, and fatty liver had all increased, compared with the values in our previous study, whereas alcoholic consumption was similar. The increase in the proportion of non-B, non-C HCC can be partially explained by a decrease in the number of HCV-related HCC cases, since the number of HCV carriers in Japan has continued to decrease. However, when the number of patients with a non-viral etiology was calculated by multiplying the proportion observed in the present study and the annual incidences of liver cancer reported by the Japanese government (data not shown), an ongoing increase was observed [19].

On the other hand, tumor characteristics and liver function did not change between the two studies. Although HCC seems to have been diagnosed at a slightly earlier stage, based on the tumor marker values and the BCLC stages, the improvement was quite small considering the progress in diagnostic procedures that has been made over the past two decades. The survival of the patients was suboptimal, although it was slightly improved compared with previous reports. The identification of a strategy for identifying individuals with a high risk of developing HCC among patients with non-viral chronic liver diseases is needed to further improve patient survival.

Fatty liver is regarded as the second largest cause of cirrhosis, after chronic viral hepatitis [20, 21]. However, the degree of fat accumulation in the liver is known to decrease with the progression of fatty liver disease [22, 23]; the final stage of this disease, burned-out NASH, is thought to be a major cause of cryptogenic cirrhosis [24, 25]. In fact, we confirmed that at the time of HCC diagnosis, fatty liver had disappeared in 7.1% of the patients in whom fatty liver had been previously observed. Considering the high prevalences of obesity, diabetes, and hypertension in this cohort, the proportion of patients with a history of fatty liver should be larger than the proportion of patients with fatty liver observed at the time of the diagnosis of HCC.

Among the various mechanisms behind obesity-associated hepatocarcinogenesis, insulin resistance and associated hyperinsulinemia are considered to be key components [26]. Therefore, sulfonylurea, which stimulates beta cells in the pancreas and insulin itself, may increase the risk of HCC [27, 28]. On the other hand, metformin reportedly decreases the risk of HCC among diabetic patients [29]. Statins, which are used to treat dyslipidemia (a major component of metabolic syndrome), also reportedly decrease the risk of HCC [30]. Angiotensin-converting enzyme inhibitors and angiotensin II receptor blockers may have a preventive effect on cancer development, including HCC [31, 32]. In the current study, we investigated the presence of prescriptions for the treatment of diabetes mellitus, dyslipidemia, and hypertension. Unfortunately, the results were unremarkable; the drug distributions were similar to those in the general population in Japan [33–35]. Clarifying the relationship between prescriptions for the treatment of these diseases and the development of HCC is likely to be difficult, since the popularity and use of different classes of drugs has been changing according to drug development, the accumulation of evidence, and revisions to treatment guidelines.

Although excessive alcohol intake is known to increase the risk of HCC in both viral-related and viral-unrelated chronic liver diseases [36], whether a longer duration of drinking increases the risk remains controversial. Corrao et al. [37] reported that the duration of alcohol intake, in addition to the daily intake, was a risk factor for cirrhosis. On the other hand, Donato et al. [38] reported that while the risk of HCC development increased according to daily alcohol consumption in a linear manner, the duration of drinking and the age at the start of drinking did not affect the risk. They also reported that former drinkers had a higher risk of HCC than current drinkers. Since their study had a case–controlled design, a bias may have existed in that patients who stopped drinking might have lived longer only to eventually develop HCC, while those who continued their excessive drinking might have died from liver failure without having survived long enough to develop HCC. In the current study, we observed an inverse relationship between the duration of drinking and the amount of daily alcohol consumption. These findings suggest that moderate drinking (e.g., daily alcohol intake between 20 and 60 g) for longer than 50 years might be a risk factor for HCC. We also showed that the GGT and AST levels decreased significantly as a result of practicing moderation in drinking. Of note, the laboratory data at the time of the diagnosis of HCC might not reflect hidden risks in the past.

Interestingly, the cubic spline hazard according to the BMI exhibited a J-curve similar to that observed in our previous study. In addition, the lowest point was also the same, at around 26 kg/m^2^. This finding is somewhat paradoxical, since a BMI > 25 kg/m^2^ is reportedly a risk factor for HCC development [39–41]. Regarding this obesity paradox, we previously reported that the skeletal muscle mass significantly affects the survival of HCC patients [42]. Patients with sarcopenia, a condition in which the skeletal muscle mass is depleted, might have been included among the underweight population, which would have reduced the overall survival rate in this group.

The increase in the BMI in the present study was relatively small, whereas the increase in the proportion of patients with obesity-related disorders was more prominent. This is probably because the median age of the present cohort was 3 years older than that in the previous study, and BMI decreases in accordance with age, reflecting reductions in muscle mass.

The present study had several limitations. First, as this study did not have a control arm for patients with viral hepatitis, we cannot estimate the impact of lifestyle-related risk factors. Second, since this study was an observational study, we cannot prove the causal relationship between the increased proportion of non-viral HCC and the increasing prevalence of obesity in Japan. However, considering the fact that alcohol consumption per capita has not increased for the last 2 decades [43] and the number of patients with diabetes is increasing in Japan [44], it is highly likely that the increasing prevalence of obesity among Japanese males has contributed to an increase in non-viral HCC.

In conclusion, the incidence of non-B, non-C HCC continued to increase during the current study period of 2011–2015. A strategy for early diagnosis needs to be established to improve patient survival. Moderate drinking for a long duration might be a risk factor for HCC. The study also reconfirmed that patients who were slightly overweight lived the longest.

## Electronic supplementary material

Below is the link to the electronic supplementary material.

Supplementary material 1 GGT, AST, and ALT values and FIB-4 indices between patients with at least 6 months of practicing moderation in drinking and controls who continued drinking, matched for gender and daily amount of alcohol intake. The difference was most prominent for GGT, followed by AST. The difference was marginally significant for ALT, and no difference in the FIB-4 indices was observed. Abbreviations: AST, aspartate aminotransferase; ALT, alanine aminotransferase; FIB-4, fibrosis-4; GGT, γ-glutamyltransferase. (TIFF 626 kb)

Supplementary material 2 Presence of prescriptions for the treatment of (A) diabetes, (B) hypertension, and (C) dyslipidemia. Abbreviations: ACEI, angiotensin-converting-enzyme inhibitors; αGI, alpha-glucosidase inhibitors; ARB, angiotensin II receptor blockers; BG, biguanide; CCB, calcium channel blockers; DIU, diuretics; DPP-4, dipeptidyl peptidase-4 inhibitors; EZT, ezetimibe; FIB, fibrates; GLN, glinides; GLP-1, glucagon-like peptide-1 agonists; ω3FA, omega-3 fatty acid; SU, sulfonylureas; SGLT-2, sodium/glucose cotransporter 2 inhibitors; TZD, thiazolidinediones. (TIFF 525 kb)

Supplementary material 3 (TIFF 388 kb)

Supplementary material 4 (TIFF 368 kb)
