# Supplementary figures and images for: A nationwide survey on non-B, non-C hepatocellular carcinoma in Japan: 2011–2015 update
Source: J Gastroenterol. 2018 Nov 29;54(4):367–76. doi: 10.1007/s00535-018-1532-5 (PMC6437291; doi:10.1007/s00535-018-1532-5)

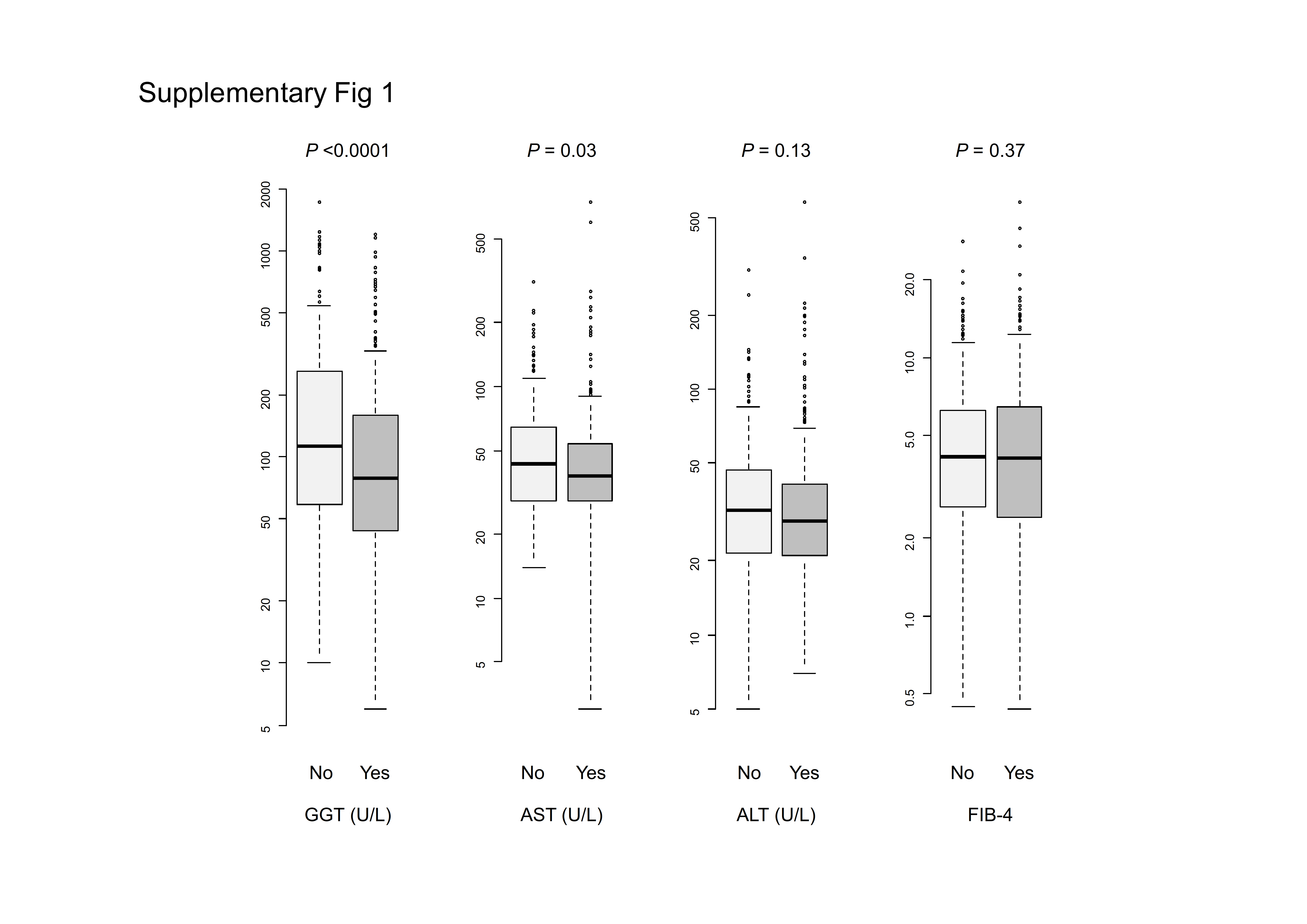

Supplement: Supplementary file 1 — Supplementary material 1 GGT, AST, and ALT values and FIB-4 indices between patients with at least 6 months of practicing moderation in drinking and controls who continued drinking, matched for gender and daily amount of alcohol intake. The difference was most prominent for GGT, followed by AST. The difference was marginally significant for ALT, and no difference in the FIB-4 indices was observed. Abbreviations: AST, aspartate aminotransferase; ALT, alanine aminotransferase; FIB-4, fibrosis-4; GGT, γ-glutamyltransferase. (TIFF 626 kb) [file 535_2018_1532_MOESM1_ESM.tif]

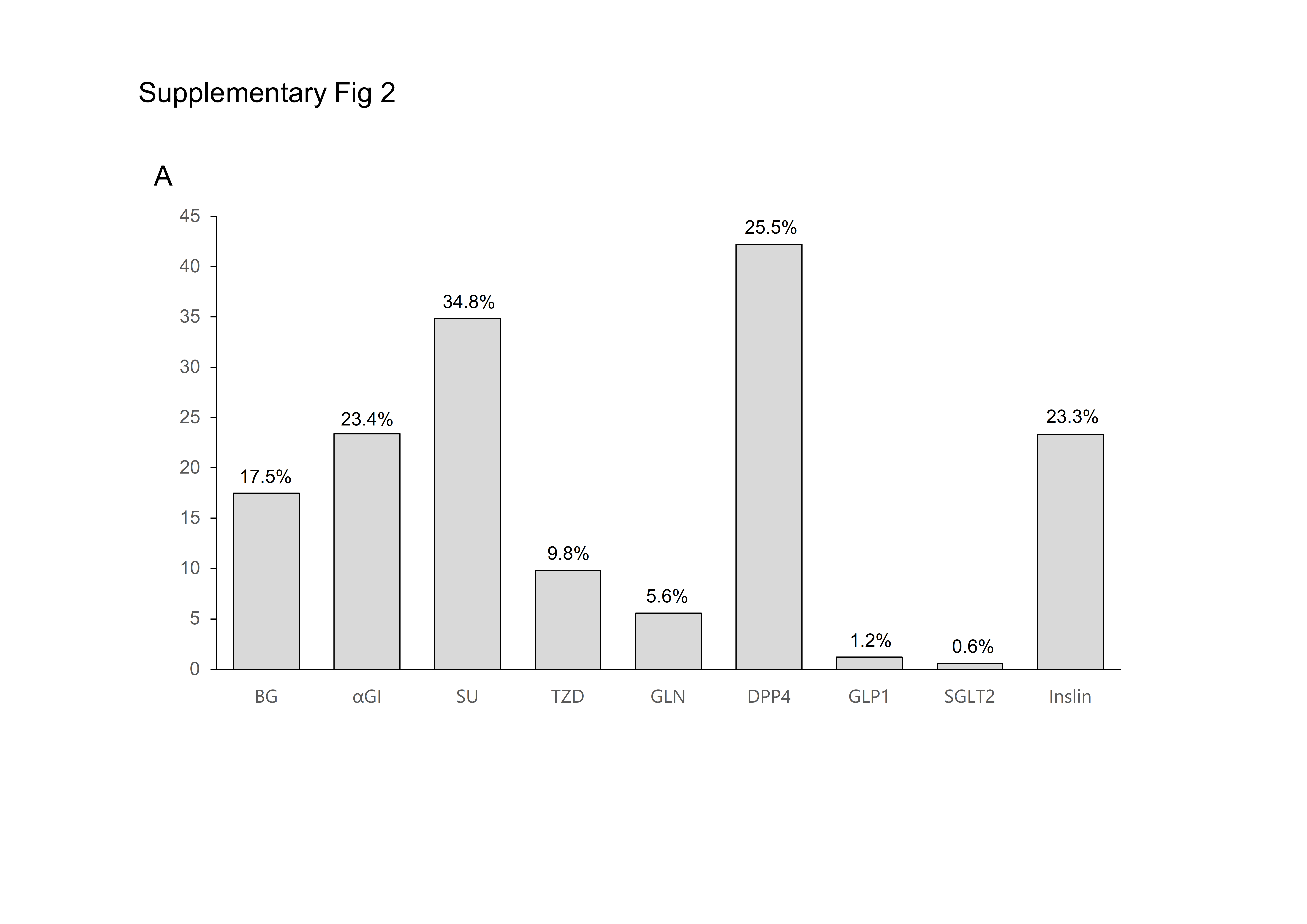

Supplement: Supplementary file 2 — Supplementary material 2 Presence of prescriptions for the treatment of (A) diabetes, (B) hypertension, and (C) dyslipidemia. Abbreviations: ACEI, angiotensin-converting-enzyme inhibitors; αGI, alpha-glucosidase inhibitors; ARB, angiotensin II receptor blockers; BG, biguanide; CCB, calcium channel blockers; DIU, diuretics; DPP-4, dipeptidyl peptidase-4 inhibitors; EZT, ezetimibe; FIB, fibrates; GLN, glinides; GLP-1, glucagon-like peptide-1 agonists; ω3FA, omega-3 fatty acid; SU, sulfonylureas; SGLT-2, sodium/glucose cotransporter 2 inhibitors; TZD, thiazolidinediones. (TIFF 525 kb) [file 535_2018_1532_MOESM2_ESM.tif]

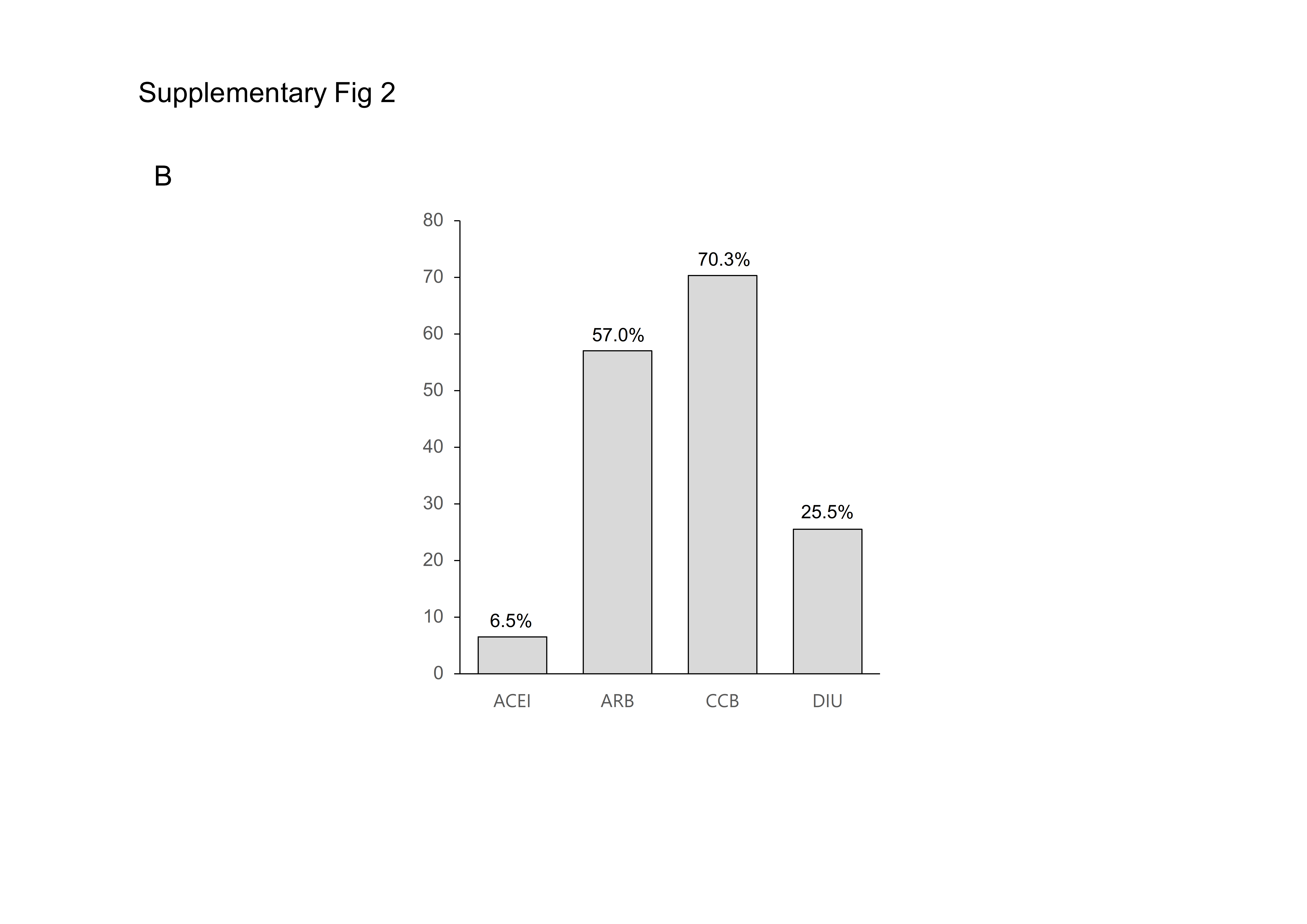

Supplement: Supplementary file 3 — Supplementary material 3 (TIFF 388 kb) [file 535_2018_1532_MOESM3_ESM.tif]

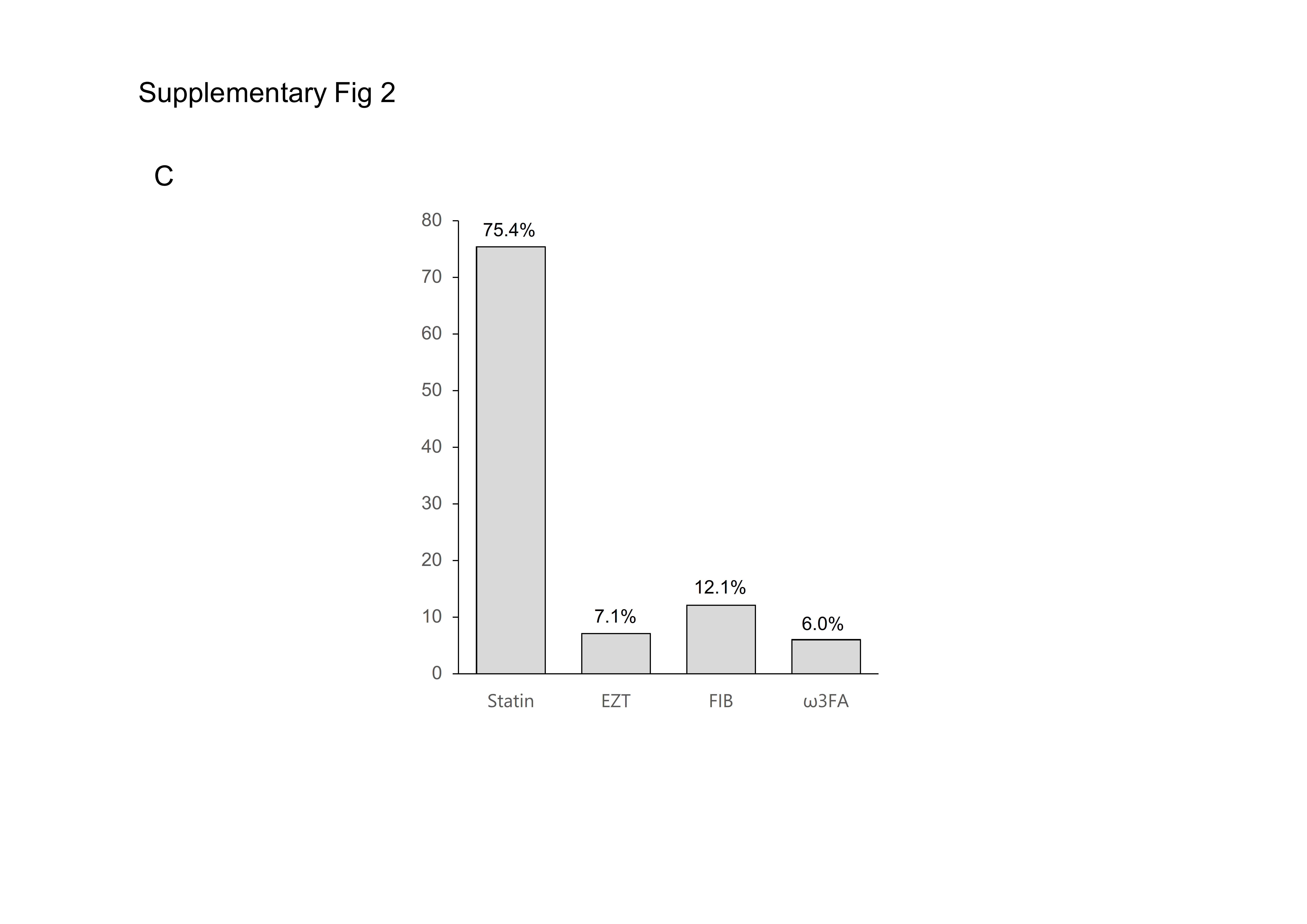

Supplement: Supplementary file 4 — Supplementary material 4 (TIFF 368 kb) [file 535_2018_1532_MOESM4_ESM.tif]
